# Supplementary material for: Phospholipid flippase ATP11C is endocytosed and downregulated following Ca2+-mediated protein kinase C activation
Source: Nat Commun. 2017 Nov 10;8:1423. doi: 10.1038/s41467-017-01338-1 (PMC5680300; doi:10.1038/s41467-017-01338-1)
Supplement: Supplementary file 1 — Supplementary Information [file 41467_2017_1338_MOESM1_ESM.pdf]

Supplementary Information

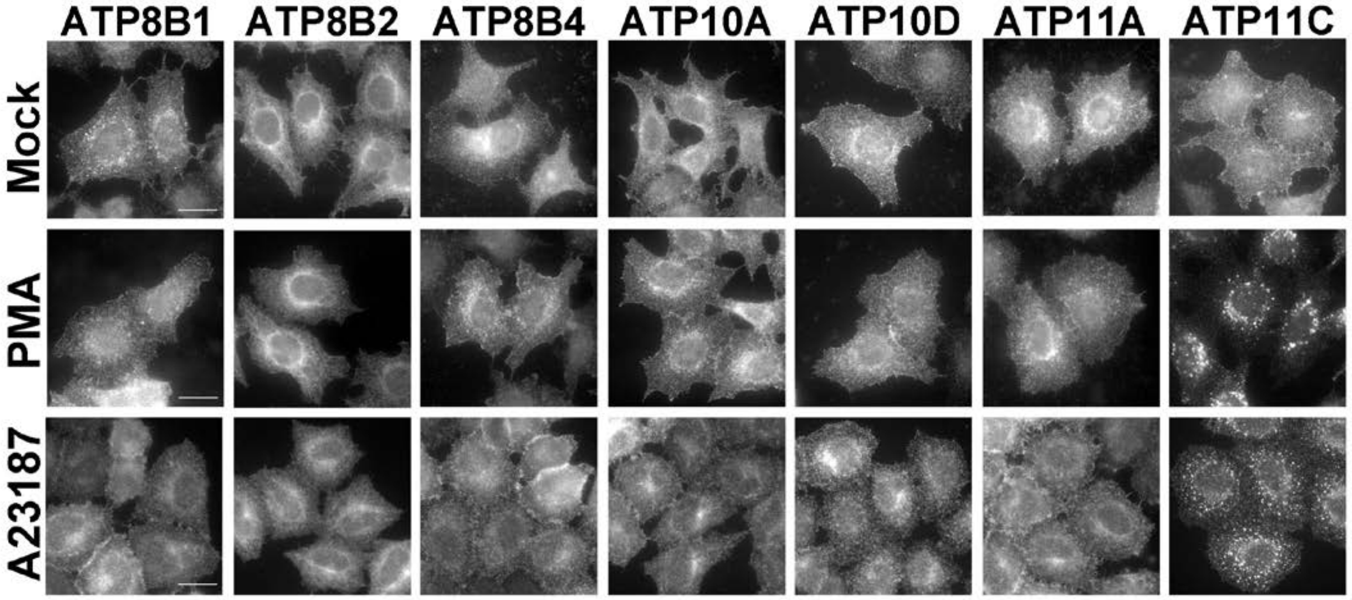

Supplementary Figure 1 Among the plasma membrane-localized P4-ATPases, only ATP11C is endocytosed upon treatment with PMA.

HeLa cells stably expressing C-terminally HA-tagged ATP8B1, ATP8B2, ATP8B4, ATP10A, ATP10D, ATP11A, and ATP11C were treated with 400 nM PMA for 15 min or with 1 μM A23187 in the presence of 1.8 mM of $CaCl_2$ (A23187), followed by fixation. The fixed cells were permeabilized and incubated with anti-HA antibody, followed by Cy3–conjugated anti-rat secondary antibody. Scale bars, 20 μm.

```
                    *  *     *****  *  **  **   *    **** **  ********** *** **********
hATP11A (1-71)  MDCSLVRTL-VHRYCAGEENWVDSRTIYVGHREPPPGAEAYIPQRYPDNRIVSSKYTFWNFIPKNLFEQFRR
hATP11C (1-69)  --MQMVPSLPPASECAGEEKRVGTRTVFVGN-HPVSETEAYIAQRFCDNRIVSSKYTLWNFLPKNLFEQFRR


                                    **             *  **    *    *  *  *  *
hATP11A (1093-1134)  -----------KKVLCRQLWPTATERVQTKSQCLSVEQSTIFMLSQTSSSLSF--
hATP11C (1089-1132)  VLKNVRRRSARRNLSCRR-----------ASDSLSARPSVRPLLLRTFSDESNVL
```

ATP11A amino acids	ATP11C amino acids	Similarity (%) ATP11A vs ATP11C	Identity (%) ATP11A vs ATP11C
1-71 (N-terminus)	1-69 (N-terminus)	69.4	58.3
72-1092	70-1088	79.7	65.6
1093-1134 (C-terminus)	1089-1132 (C-terminus)	27.3	18.2

Supplementary Figure 2 Sequence comparisons between ATP11A and ATP11C

a. Sequence alignments of N-terminus or C-terminus of ATP11A and ATP11C by EMBOSS Needle [1]. Di-leucine-like signals are underlined.

b. Sequence identities and similarities between each region of ATP11A and ATP11C.

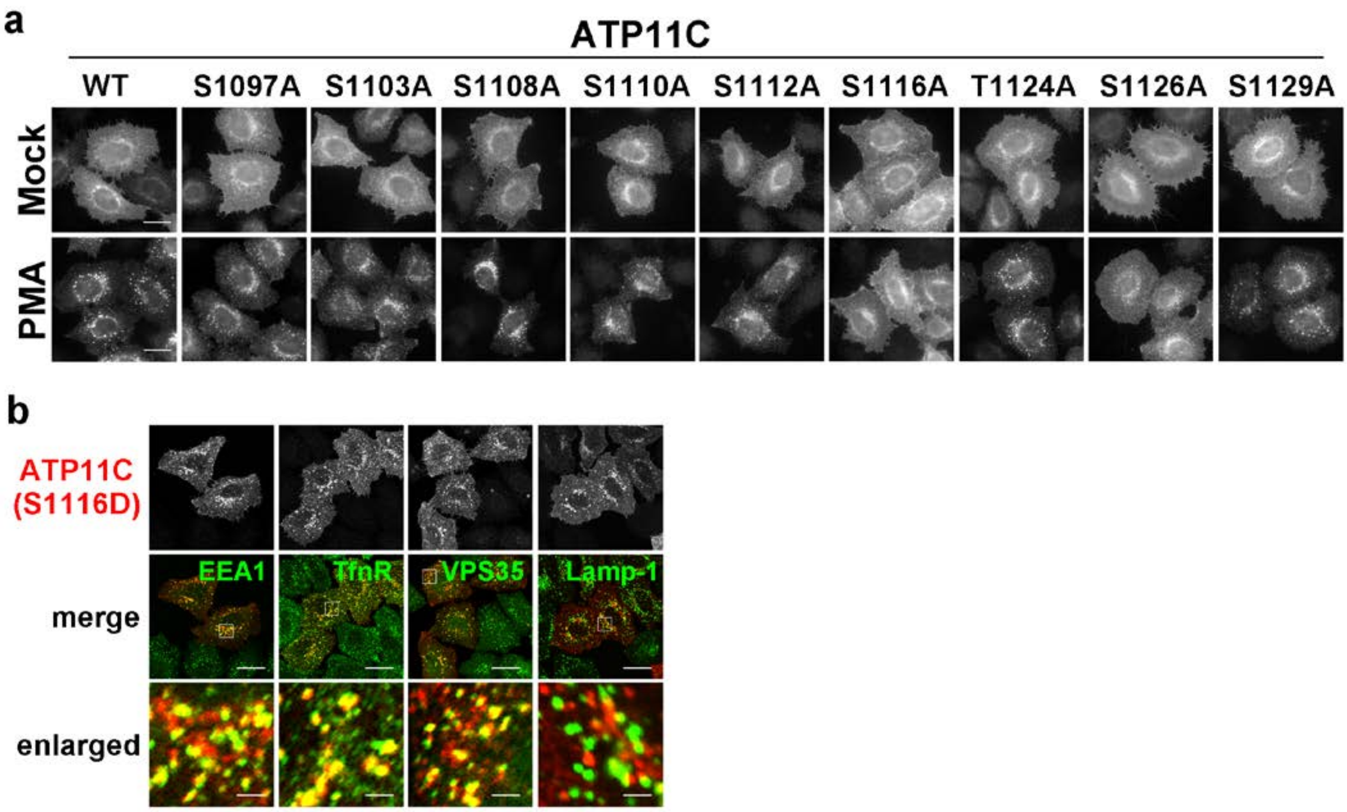

Supplementary Figure 3 Localization of C-terminus point mutants of ATP11C.

a. HeLa cells were transiently co-transfected with expression vectors for FLAG-tagged CDC50A and wild type or point mutants of HA-tagged ATP11C in which nine Ser/Thr residues of C-terminal cytoplasmic region of ATP11C were replaced individually with Ala. Cells were treated with DMSO (Mock) or 400 nM PMA, followed by fixation. The fixed cells were then incubated with anti-HA antibody, followed by Cy3–conjugated anti-rat secondary antibody. Scale bars, 20 μm.

b. HeLa cells were transiently co-transfected with expression vectors for FLAG-tagged CDC50A and the HA-tagged S1116D mutant of ATP11C. Cells were fixed and doubly stained for HA, and EEA1, TfnR, VPS35, or Lamp-1, followed by incubation with Cy3–conjugated anti-rat and Alexa Fluor 488–conjugated anti-mouse or anti-goat secondary antibodies. Images were obtained by confocal microscopy. Scale bars, 20 μm. Insets were enlarged (bars, 2 μm).

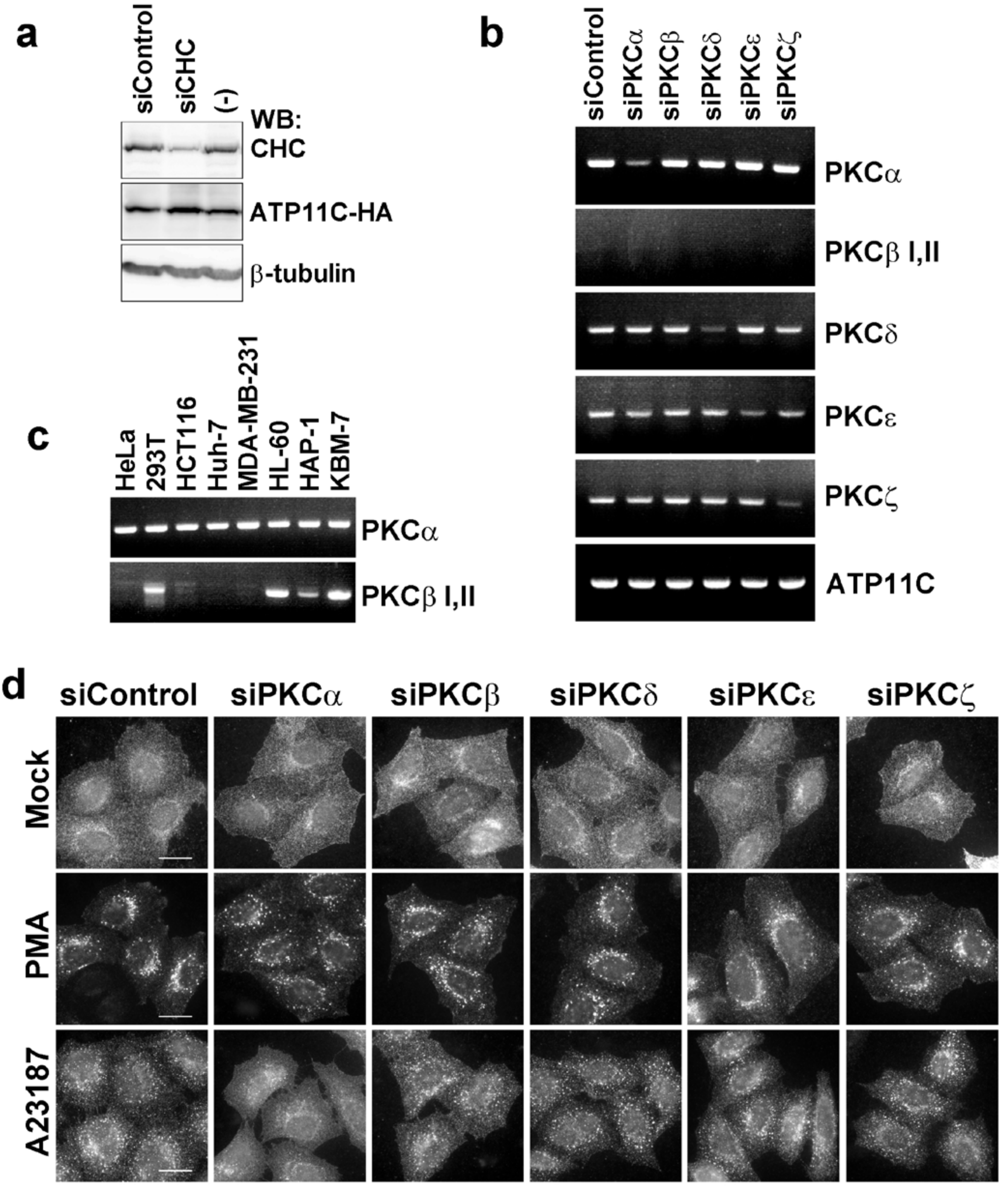

Supplementary Figure 4 Knockdown of clathrin heavy chain and each isoform of PKC in HeLa cells stably expressing ATP11C.

a. HeLa cells stably expressing ATP11C-HA were transfected with non-targeting siRNA (siControl), or siRNA against clathrin heavy chain (siCHC). Each cell lysate was analyzed by immunoblotting with

anti-CHC, anti-HA, and anti-β-tubulin antibodies ((-), non-transfected cell lysate).

b. HeLa cells stably expressing ATP11C-HA were transfected with non-targeting siRNA (siControl), or siRNA against the indicated PKC isoforms. Total RNA was isolated from the cells and subjected to RT-PCR.

c. RT-PCR was performed using total RNA isolated from the indicated human cell lines. PKCβ was not expressed in HeLa cells.

d. HeLa cells treated with the indicated siRNAs were treated for 15 min with DMSO (Mock), 400 nM PMA (PMA), or 1 μM A23187 in the presence of $CaCl_2$ (A23187), followed by fixation. The fixed cells were permeabilized and incubated with anti-HA antibody, followed by Cy3–conjugated anti-rat secondary antibody. Endocytosis of ATP11C upon treatment with A23187 was inhibited in cells depleted of PKCα, but not other PKC isoforms. Scale bars, 20 μm.

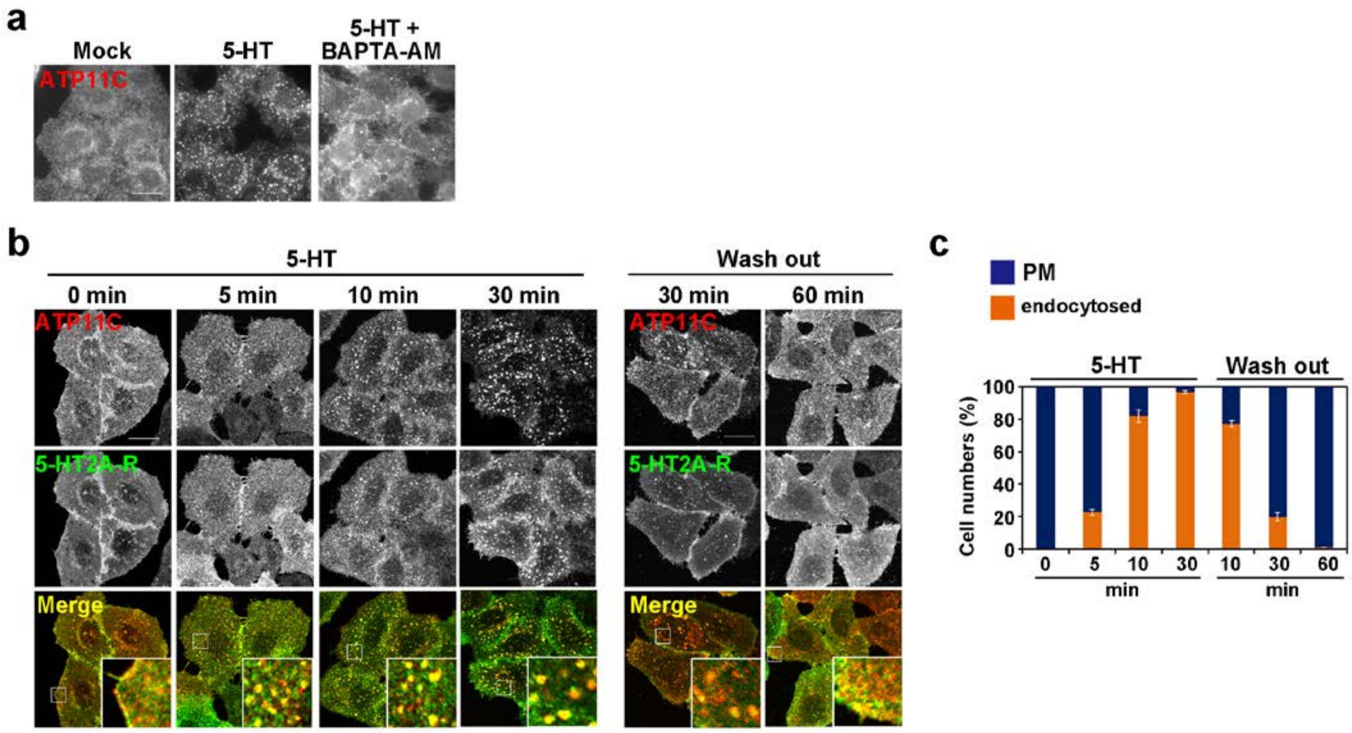

Supplementary Figure 5 ATP11C is endocytosed by treatment with serotonin.

a. HeLa cells stably expressing C-terminally HA-tagged ATP11C and untagged 5-HT2A-R were serum starved for 2 h and pretreated with 50 μM BAPTA-AM for 1hr before treatment with 500 nM 5-HT for 15 min. **b**. HeLa cells stably expressing C-terminally HA-tagged ATP11C and C-terminally FLAG-tagged 5-HT2A-R were serum starved for 3 h and treated with 500 nM of 5-HT for indicated times (5-HT). After 30 min treatment, cells were washed with 5-HT-free medium and incubated in the medium for indicated times (wash out). **c**. Cells in which ATP11C localized to the plasma membrane (PM), or to the plasma membrane and endosomes (endocytosed) were counted; counts were normalized against the total number of counted cells. In each sample, 516–698 cells were counted. Graphs display averages ± SD from four independent experiments.

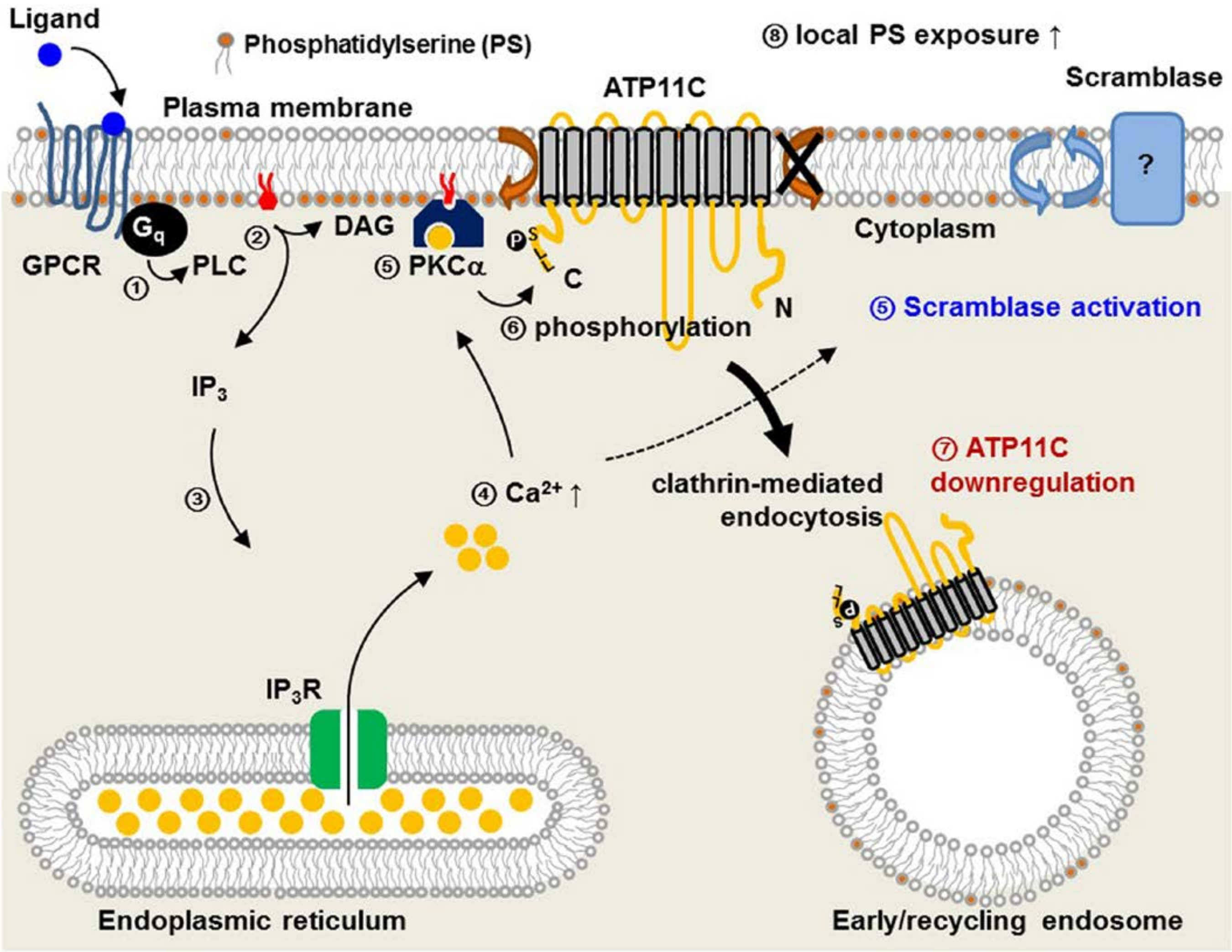

Supplementary Figure 6 Model for local PS exposure in response to cellular signaling.

At steady state, PS is mainly distributed in the cytoplasmic leaflet of the plasma membrane by action of ATP11C. Cytosolic Ca^{2+} increases in response to cellular signaling such as Gq-coupled receptor-mediated signal transduction (1). (2) Activated phospholipase C hydrolyzes phosphatidylinositol 4,5-bisphosphate to inositol 1,4,5-trisphosphate (IP_3) and diacylglycerol (DAG). (3)(4) IP_3 stimulates a rise in cytosolic Ca^{2+}. (5) Ca^{2+}-dependent scramblase is activated, and trans-bilayer lipid asymmetry is abolished (dashed line). (5) An increase in Ca^{2+} and DAG leads to activation of PKCα (solid line). (6) Phosphorylation of the C-terminal region of ATP11C by PKCα results in generation of a functional di-leucine motif. (7) ATP11C is downregulated by clathrin-mediated endocytosis. (8) Scrambled PS cannot be flipped into the inner leaflet; consequently, local PS exposure is maintained.

Western blots of Figure 1b

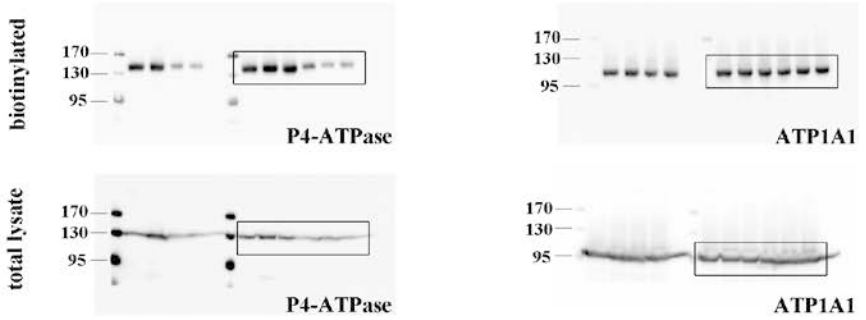

Western blots of Figure 8c

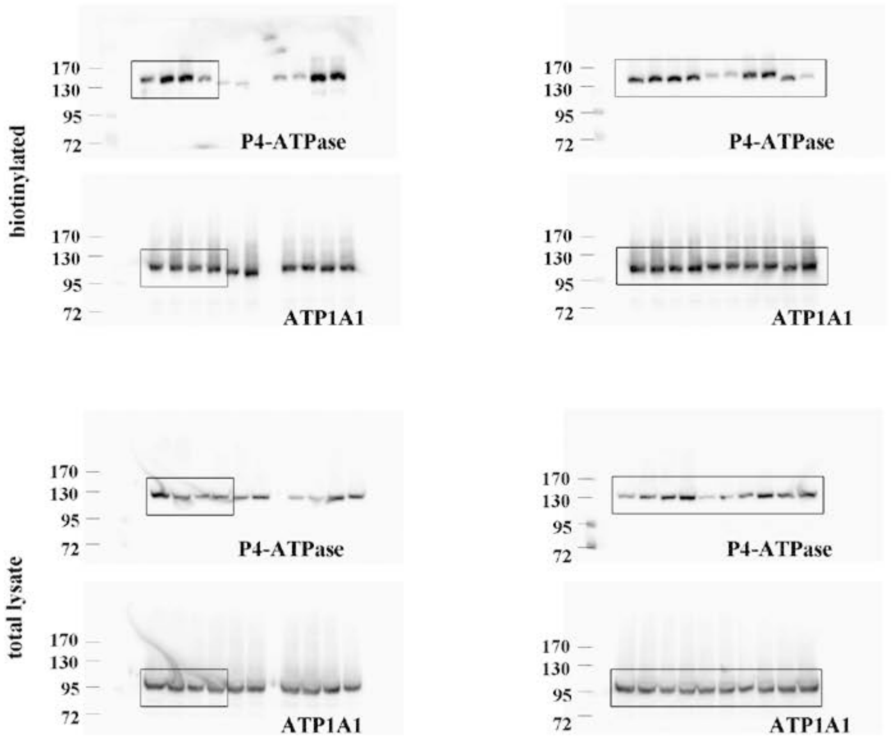

Western blots of Supplementary Figure 4a

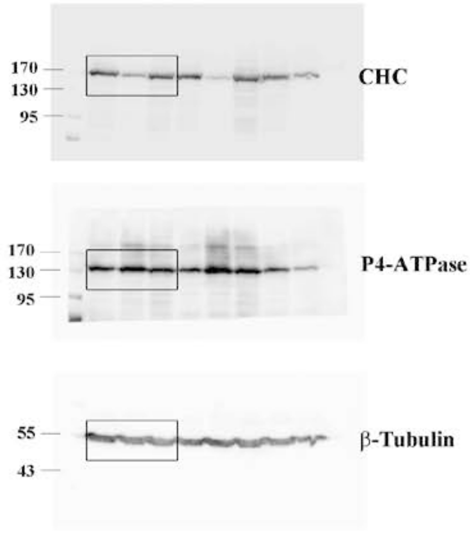

Supplementary Figure 7 Full scans of Western blots shown in Figures 1b and 8c, and Supplementary Figure 4a.

Supplementary Table 1 Oligos used in this study.

**Primer**	**Sequence**	**Use**
hATP11C-NS	GCGGATCCACCatgcagatggtcccatctctcc	ATP11C
hATP11C-CAS2	CCGCTCGAGAGtacattagattcgtctgagaatg	ATP11C
hATP11A-NS	GCGGATCCAccatggactgcagcctcgtg	ATP11A
hATP11A-CAS2	CCTGTCGACGCgaaactcaggctgctggaag	ATP11A
hATP11C-N-Bln-S	ggccctaggaatctgtttgaacagtttagaag	ATP11ACC
hATP11A-N-Bln-AS	ccgcctaggtataaagttccaaaatgtgtac	ATP11ACC
hATP11A-N-Bln-S	ggccctaggaatttatttgaacaattcagaag	ATP11CAA
hATP11C-N-Bln-AS	ccgctcgagctacctagggagaaaattccaaagtgtatac	ATP11CAA
hATP11A-3360S	GCCGATATCctcaagaaagtcctgtgcc	ATP11CCA
hATP11C-C-EcoRV-AS	ggcgatatctaatactatcagaagaatctcagg	ATP11CCA
11AAC-CS1	CTCAAGAAAGTCCTGaagaatgtaagaagaagaagtgcc	ATP11AAC
11AAC-CAS2	CTAGATATCTCGAGAGtacattag	ATP11AAC
hATP11C-S1097AD-S	gtattaaagaatgtaagaagaagaGMtgccaggagaaatctgagctg	S1097 mutagenesis
hATP11C-S1097AD-AS	cagctcagatttctcctggcaKCtcttcttcttacattctttaatac	S1097 mutagenesis
hATP11C-S1103AD-S	gaagaagtgccaggagaaatctgGMctgtagaagggcatctgactc	S1103 mutagenesis
hATP11C-S1103AD-AS	gagtcagatgcccttctacagKCcagatttctcctggcacttcttc	S1103 mutagenesis
hATP11C-S1108A-S	atctgagctgtagaagggcaGctgactcattatccgccagac	S1108 mutagenesis
hATP11C-S1108A-AS	gtctggcggataatgagtcagCtgcccttctacagctcagat	S1108 mutagenesis
hATP11C-S1110A-S	gctgtagaagggcatctgacGcattatccgccagaccttcag	S1110 mutagenesis
hATP11C-S1110A-AS	ctgaaggtctggcggataatgCgtcagatgcccttctacagc	S1110 mutagenesis
hATP11C-S1112A-S	gtagaagggcatctgactcattaGccgccagaccttcagtcag	S1112 mutagenesis
hATP11C-S1112A-AS	ctgactgaaggtctggcggCtaatgagtcagatgcccttctac	S1112 mutagenesis
hATP11C-S1116A-S	gactcattatccgccagacctGcagtcagacctcttcttttacg	S1116 mutagenesis
hATP11C-S1116A-AS	cgtaaaagaagaggtctgactgCaggtctggcggataatgagtc	S1116 mutagenesis
hATP11C-S1116D-S	gactcattatccgccagacctGATgtcagacctcttcttttacgaac	S1116 mutagenesis
hATP11C-S1116D-AS	gttcgtaaaagaagaggtctgacATCaggtctggcggataatgagtc	S1116 mutagenesis
hATP11C-T1124AE-S	cagtcagacctcttcttttacgaGMAttctcagacgaatctaatgtactc	S1124 mutagenesis
hATP11C-T1124AE-AS	gagtacattagattcgtctgagaaTKCtcgtaaaagaagaggtctgactg	S1124 mutagenesis
hATP11C-S1126AD-S	cagacctcttcttttacgaacattcGMTgacgaatctaatgtactctcgag	S1126 mutagenesis
hATP11C-S1126AD-AS	ctcgagagtacattagattcgtcAKCgaatgttcgtaaaagaagaggtctg	S1126 mutagenesis
hATP11C-S1126AE-S	cagacctcttcttttacgaacattcGMAgacgaatctaatgtactctcgag	S1126 mutagenesis
hATP11C-S1126AE-AS	ctcgagagtacattagattcgtcTKCgaatgttcgtaaaagaagaggtctg	S1126 mutagenesis
hATP11C-S1129AD-S	cttttacgaacattctcagacgaaGMTaatgtactctcgagatatctagac	S1129 mutagenesis
hATP11C-S1129AD-AS	gtctagatatctcgagagtacattAKCttcgtctgagaatgttcgtaaaag	S1129 mutagenesis
hATP11C-L1120A-S	ccagaccttcagtcagacctGCtcttttacgaacattctcagac	L1120 mutagenesis
hATP11C-L1120A-AS	gtctgagaatgttcgtaaaagaGCaggtctgactgaaggtctgg	L1120 mutagenesis
hATP11C-L1121A-S	cagaccttcagtcagacctcttGCtttacgaacattctcagacgaatc	L1121 mutagenesis
hATP11C-L1121A-AS	gattcgtctgagaatgttcgtaaaGCaagaggtctgactgaaggtctg	L1121 mutagenesis
hATP11C-L1122A-S	gaccttcagtcagacctcttcttGCacgaacattctcagacgaatc	L1122 mutagenesis
hATP11C-L1122A-AS	gattcgtctgagaatgttcgtGCaagaagaggtctgactgaaggtc	L1122 mutagenesis

hATP11C-C-9Ala	GGCAGATCTAGAgtattaaagaatgtaagaagaagaGCtgccaggagaaatctgGCctgtagaagggcaGctgacGcattaGccgccagacctGcagtcagacctcttcttttacgaGcattcGcagacgaaGctaatgtattgtaaCTCGAGCGG	9-Ala mutagenesis
hATP11C-C-Ser1116	GGCAGATCTAGAgtattaaagaatgtaagaagaagaGCtgccaggagaaatctgGCctgtagaagggcaGctgacGcattaGccgccagacctTcagtcagacctcttcttttacgaGcattcGcagacgaaGctaatgtattgtaaCTCGAGCGG	8-Ala(S1116) mutagenesis
hATP11C-C-Ser1126	GGCAGATCTAGAgtattaaagaatgtaagaagaagaGCtgccaggagaaatctgGCctgtagaagggcaGctgacGcattaGccgccagacctGcagtcagacctcttcttttacgaGcattcTcagacgaaGctaatgtattgtaaCTCGAGCGG	8-Ala(S1126) mutagenesis
hATP11C-3334AS	cctggcacttcttcttcttacattc	9-Ala, 8-Ala(S1116), and 8-Ala(1126)
11C-pENTR-S	ctcgagatatctagacccagct	9-Ala, 8-Ala(S1116), and 8-Ala(1126)
hATP11C-3364S	gtattaaagaatgtaagaagaaga	9-Ala, 8-Ala(S1116), and 8-Ala(1126)
hATP11C-SA-pENTR	gctgggtctagatatctcgagagtacattagcttcgtctg	9-Ala, 8-Ala(S1116), and 8-Ala(1126)
11C-BsrGI-S	GCCTGTACAAGgtattaaagaatgtaagaagaag	CT-WT, CT-9-Ala, CT-8-Ala/S1116, and CT-8-Ala/S1126
11C-C-AS	ccgctcgagttacaatacattag	CT-WT, CT-9-Ala, CT-8-Ala/S1116, and CT-8-Ala/S1126
pENTR-IF-NS	cttaaccatggtcgactggatcc	7-Ala(S1116/1126)
Ser1126-SLICE-AS	ctgagaatgCtcgtaaaagaag	7-Ala(S1116/1126)
Ser1126-SLICE-S	cttttacgaGcattctcagacgaaGctaatgtaCTCTC	7-Ala(S1116/1126)
hPKCα-854S	ggccagtggatggtacaagttg	RT-PCR
hPKCα-1117AS	ctgtcggcaagcatcacctttc	RT-PCR
hPKCβ-998S	gaaagccagtgttgatggctg	RT-PCR
hPKCβ-1286AS	gccttttcgttctgaaagcatg	RT-PCR
hPKCδ-1135S	gtgaagactgcggcatgaatg	RT-PCR
hPKCδ-1449AS	caagcagcaccttcccgaag	RT-PCR
hPKCε-536S	cgaggactggattgatctggag	RT-PCR
hPKCε-814AS	caggtgcagacttgacactgg	RT-PCR
hPKCζ-578S	caggagagcgtactgcggtc	RT-PCR
hPKCζ-861AS	ctggcttaaggtcctccgag	RT-PCR
hPKCγ-801S	ccacgtaactgttggcgaggc	RT-PCR
hPKCγ-1110AS	cttgtaccagccatccacggg	RT-PCR
m5HT2A-NS	GCCGGATCCACCatggaaattctctgtgaagacaatatctc	mouse 5HT2A
m5HT2A-CAS1	CGCGTCGACtcacacacagctaaccttttcattcacgg	mouse 5HT2A
m5HT2A-CAS2	CGCGTCGacacacagctaaccttttcattcacgg	mouse 5HT2A stop

Supplementary Table 2 Antibodies used in this study.

Antibody	Supplier	Catalogue number	Dilution for immunofluorescence	Dilution for immunoblotting
anti-HA (3F10)	Sigma-Aldrich	11867423001	1:500	1:500
anti-DYKDDDDK (1E6)	WAKO	018-22381	1:500	
anti-ATP1A1 (EP1845Y)	Abcam	ab76020	1:2000	1:2000
anti-EEA1 (14)	BD Biosciences	BDB610457	1:500	
anti-transferrin receptor (H68.4)	Thermo Fisher Scientific	13-6800	1:1000	
anti-VPS35	Novus Biologicals	NB100-1397	1:500	
anti-Lamp1 (H4A3)	BD Biosciences	555798	1:2000	
anti-clathrin heavy chain (23)	BD Biosciences	610500		1:500
anti-clathrin heavy chain (X22)	Thermo Fisher Scientific	MA1-065	1:500	
anti-β-tubulin (KMX-1)	Merck	MAB3408		1:1000

Supplementary Reference

1. Rice, P., Longden, I. & Bleasby, A. EMBOSS: the European Molecular Biology Open Software Suite. *Trends Genet* **16**, 276-277 (2000).
