# Supplementary material for: Phospholipid flippase ATP11C is endocytosed and downregulated following Ca2+-mediated protein kinase C activation
Source: Nat Commun. 2017 Nov 10;8:1423. doi: 10.1038/s41467-017-01338-1 (PMC5680300; doi:10.1038/s41467-017-01338-1)
Supplement: Supplementary file 3 — Description of Additional Supplementary Files [file 41467_2017_1338_MOESM3_ESM.pdf]

Description of Additional Supplementary Files

File Name: Supplementary Movie 1
Description: Time-lapse imaging of EGFP-ATP11C following treatment with PMA. HeLa cells transiently expressing EGFP-ATP11C were subjected to time-lapse recording. Cells were treated with 400 nM PMA starting at 5 min of recording time. Images were acquired sequentially every 7.8 seconds. Movie corresponds to 20 frames per second

File Name: Supplementary Movie 2
Description: Time-lapse imaging of EGFP-ATP11C following treatment with serotonin. HeLa cells transiently expressing EGFP-ATP11C and 5-HT2A-R were subjected to time-lapse recording. Cells were treated with 500 nM 5-HT starting at 5 min of recording time. Images were acquired sequentially every 8.0 seconds. Movie corresponds to 20 frames per second
